# The application of lasers in vital pulp therapy: a review of histological effects

**DOI:** 10.1007/s10103-023-03854-7

**Published:** 2023-09-21

**Authors:** Farzaneh Afkhami, Golriz Rostami, Chun Xu, Laurence J. Walsh, Ove A. Peters

**Affiliations:** 1 School of Dentistry, The University of Queensland, 288 Herston Road, Brisbane, QLD 4006 Australia; 2 Department of Endodontics, School of Dentistry, Tehran University of Medical Sciences, Tehran, Iran; 3 Laser Research Center of Dentistry, Dentistry Research Institute, Tehran University of Medical Sciences, Tehran, Iran

**Keywords:** Dental pulp, Dental pulp capping, Histology, Lasers

## Abstract

Vital pulp therapy (VPT) is primarily intended to preserve the vitality of pulp tissues, which have been exposed for any reason. Various materials and techniques have been proposed to improve treatment outcomes, including the use of lasers. This study aimed to review the histological results of different dental lasers including low-level lasers, carbon dioxide (CO_2_), erbium-doped yttrium aluminum garnet laser (Er:YAG), neodymium-doped yttrium aluminum garnet (Nd:YAG), erbium, chromium:yttrium-scandium-gallium-garnet (Er,Cr:YSGG) lasers, and diode lasers for VPT. This focused review included a comprehensive electronic search of Scopus, MEDLINE, Web of Science, and Google Scholar databases from 2000 to 2022 by two independent investigators. Different combinations of keywords were used, and reference mining of related papers was done. The review included studies related to histologic evaluation of laser-assisted vital pulp therapy that stated the laser parameters that were used. Articles with radiographic or clinical assessments or articles lacking necessary data were excluded. Non-English articles were excluded unless their abstract was in English and encompassed the necessary data. Most studies indicated the efficacy of lasers for reduction of inflammation, acceleration of healing, and increasing the thickness of dentinal bridge. According to the evidence, lasers used in combination with pulp capping agents are beneficial to enhance the success rate of VPT.

## Introduction

Vital pulp therapy (VPT) is performed to preserve the vitality of the pulp tissue exposed due to caries, trauma, or restorative procedures [1]. Although some studies have recommended that VPT should be performed primarily or exclusively for young patients, more recent investigations and position papers on the open or closed status of the tooth apex and age of patients have suggested that VPT can also be performed for permanent teeth and patients up to 60 years of age, or even older [2–4].

Preservation of pulp vitality instead of conventional root canal therapy is desirable because it has been well documented that endodontically treated teeth have a lower survival rate than teeth with a vital pulp [5]. Among the reasons cited for this are the loss of tooth structure, inability to produce reparative dentine, and absence of regenerative capacity in non-vital teeth [4, 6, 7]. The main goal of VPT is to preserve pulp vitality, in order to maintain and regenerate the dental pulp complex [7].

Currently, not only primary teeth, but also permanent teeth with an immature or mature apex, and those with reversible pulpitis, may also be candidates for VPT. According to a recently published concept, teeth that formerly would have been treated by pulpectomy and root canal therapy due to irreversible pulpitis may now undergo less invasive procedures such as VPT [8, 9]. According to histological studies, there is no distinct boundary between reversible and irreversible pulpitis. Instead, inflammation of the pulp may be categorized as initial, mild, moderate, and severe [8, 9]. Since no specific clinical test can determine in real time the actual extent of inflammation, direct observation of the pulp tissue under a microscope is considered the best strategy to clinically understand pulpal conditions [9, 10]. Accordingly, the exposed area is inspected under a microscope and if healthy pulp tissue is not observed or bleeding cannot be stopped after a predetermined time (2–5 min), another layer of pulp tissue is removed, and the remaining pulp is inspected again under the microscope. This process is then repeated until a healthy wound appearance is observed, and hemostasis can be achieved. Thus, it is the histological pulp tissue status, and not the clinical diagnosis, that determines extent of tissue removal or the type of VPT procedures that are performed [9, 10].

The following VPT procedures are considered, and the choice of these depends on the extent of pulpal inflammation:

- **Indirect pulp capping:** In this procedure, the deepest layer of carious dentine is coated with biocompatible and bioactive materials. It is believed that the bacteria present in this residual thin layer of caries are well sealed and inactivated by medicaments and restorative materials, providing the pulp with an opportunity to resume its reparative activity [11]. Indirect pulp capping is the treatment of the dentine close to the pulp which on the basis of recent contradictory definitions and guidelines might be either caries free or carious [12]. While according to the recent statement from the American Association of Endodontists, complete removal of caries is indicated for VPT [9]. Due to the possibility of spread of microorganisms during cavity preparation, the dentine close to the pulp can be disinfected as well as the whole cavity using sodium hypochlorite, chlorhexidine [12] or lasers [13], after which an antimicrobial capping material is placed [12].
- **Direct pulp capping**: Direct pulp capping is performed when the exposed pulp tissue is apparently healthy, and its bleeding can be controlled easily [10]. In this process, the exposed pulp is sealed with a biocompatible material. This technique attempts to preserve the vitality of the exposed pulp, and to induce a reparative pulpal response that forms reparative dentine and protects the pulp against further bacterial contamination [14]. In this procedure, a small portion of the pulp is exposed, and the volume of remaining pulpal tissue is greater than for other VPT approaches. This aspect may be important in the formation of the dentinal bridge [15]. Results regarding the efficacy of direct pulp capping in primary teeth are controversial [11].
- **Pulpotomy:** A pulpotomy can be partial or complete. In a partial or Cvek pulpotomy, pulp tissue is removed to a depth of approximately 2 mm, and the remaining coronal and radical pulp is sealed with a biocompatible material [16]. This procedure is similar in principle to direct pulp capping, but there is a smaller volume of residual pulp tissue than with direct pulp capping [15]. Depending on the extent of pulpal inflammation, complete pulpotomy may be required, where all coronal pulp tissue is removed. Subsequently, the radicular pulp is sealed with different materials or techniques [1].

Lasers emit coherent, monochromatic collimated light. The absorption characteristics of laser energy in tissue depend on the wavelength [17]. Major types of lasers used in dentistry are the neodymium:yttrium-aluminum garnet (Nd:YAG), carbon dioxide (CO_2_), argon, erbium:yttrium aluminum garnet (Er:YAG), erbium, chromium:yttrium-scandium-gallium garnet (Er,Cr:YSGG), and diode lasers [18]. In endodontics, lasers are used for pulpal diagnosis, disinfection, shaping of root canals, and vital pulp therapy [18]. Laser therapy has recently gained attention as a non-pharmaceutical approach for VPT. Although several materials and techniques have been used to date for VPT, uncertainty still exists regarding their success rate [4, 19]. Laser irradiation of exposed pulp tissue has been undertaken using many laser types, starting with the CO_2_ laser, with the aim of inducing dentinal bridge formation [20]. Laser application as an adjunct for VPT has been studied to explore its antibacterial, bio-stimulation, hemostatic, and wound healing actions [20, 21], and its ability to increase the expression of lectin and collagen [22].

- **CO**_**2**_**:** The most common CO_2_ laser wavelengths are 9300 and 10,600-nm, and the laser can be operated in continuous-wave, gated pulsed, or super-pulsed modes [17]. These far infrared wavelengths are well absorbed by water, and therefore these lasers are useful for soft tissue surgery as they ablate tissue rapidly and can be used as an alternative to surgical scalpels. Optimal hemostasis occurs due to thermal effects which seal small blood vessels [23]. When applied on surfaces with low water content but high levels of apatite mineral, carbonization and fusion may occur [17].
- **Er:YAG:** Er:YAG laser emits infrared radiation at 2940-nm wavelength and typically operates in free running pulsed mode [17]. Since this wavelength matches the middle infrared absorption peak of water, this laser can ablate enamel and dentine by a micro explosive process [24]. Dentine has greater absorption and faster ablation than enamel because of its higher water content, and likewise deciduous teeth ablate faster than permanent teeth because their components contain more water [25]. The Er:YAG laser can ablate enamel and dentine, as well as soft tissues with minimal thermal side effects [17, 24, 26]. When used for bleeding control, pulpal blood flow promptly and reversibly decreases for a limited time (3–6 min), and no hyperemic reaction occurs due to heat [26].
- **Er,Cr:YSGG:** Er,Cr:YSGG laser energy has a wavelength of 2780-nm. Its effects are similar to those of the Er:YAG laser, and it can ablate tooth structure [17]. Bleeding can be effectively controlled by this laser with long duration pulses that absorb in hemoglobin [20]. The hemostatic ability of both the Er,Cr:YSGG and Er:YAG lasers is less than that of the CO_2_ laser and of diode lasers [27]. The Er,Cr:YSGG laser exerts similar actions to the Er:YAG laser in terms of forming dentinal bridges and reparative dentine [20].
- **Nd:YAG:** This laser emits near infrared light at 1064-nm wavelength, and can operate in continuous wave mode as well as free running pulsed mode [17]. It is well absorbed by hemoglobin, and the accompanying thermal effects give excellent hemostasis [13]. This laser can be used for soft tissue surgical procedures, [28] as well as photothermal disinfection and coagulation. These capabilities are relevant to VPT [29].
- **Diode:** The wavelength emitted by semiconductor diode lasers depends on their construction, with common wavelengths being GaAlAs lasers at 810-nm wavelength, and InGaAs at 940 and 980-nm [17]. Diode lasers can operate in continuous wave, gated pulse, and super-pulsed modes. They are used widely for soft tissue surgery, where they give excellent hemostasis [30]. The smaller size, ease of handling and set-up, and lower cost are among their advantages over other laser types [30]. Diode laser energy in the near infrared region gives coagulation and antimicrobial effects, but has little absorption in dental hard tissues [31, 32]. Thus, soft tissue surgery can be performed safely adjacent to dental hard tissues [33]. Diode lasers in the 940–980-nm range are suitable for VPT, as there is modest absorption in water at 980 nm [34], and in dental pulp tissue [31]. Diode lasers are used in contact mode with a hot tip, and the soft tissue adjacent to the tip is affected, but not adjacent hard tissues [34].
- **Low-level laser therapy (LLLT):** Also known as photobiomodulation (PBM), this approach uses laser energy in the visible red (600–680 nm) or near infrared regions (700–940 nm) with low average powers (less than 500 mW) in continuous wave or pulsed modes, and a relatively low energy density, ranging from 0.04 to 50 J/cm^2^ [35] but typically not more than 15 J/cm^2^. While helium-neon lasers were originally used for this purpose, they have been replaced gradually with diode lasers from 635 to 830 nm [36]. Lasers with wavelengths from 600 to 700 nm in the visible spectrum, and energy densities from 0.5 to 4.0 J/cm^2^ can promote cellular proliferation [35]. Unlike laser therapies which operate on ablative or thermal mechanisms, PBM uses photochemical mechanisms, where the light acts on cells to stimulate mitochondrial enzyme systems to achieve a PBM effect [37]. Since laser outputs are typically low, there are no issues with heat, sound, or vibration [35]. However, if excessively high average powers are used with any laser type, then significant impacts on temperature at the level of the pulp chamber can occur. As an example, in one study, the average power was 417 mW, and this increased the temperature above the 5.5 °C threshold for the maximum temperature rise tolerated by pulp tissue [38]. Beyond this threshold, pulpal damage, degeneration of odontoblasts, and pulpal death are more likely [38]. Therefore, irradiation parameters should be clinically optimized for each laser type and its dental application.

The photochemical mechanism of action for PBM does not rely on thermal effects [37], unlike the CO_2_ and Nd:YAG lasers, which cause temperature elevations in the target tissue [35]. PBM can induce tissue remodeling, acceleration of wound healing processes by increasing the production of fibroblast growth factor, and at the same time decreasing the production of pro-inflammatory mediators [15]. Moreover, PBM affects tissue vasculature, by causing vasodilation of capillaries, thus promoting local circulation, and raising the level of tissue oxygenation, resulting in a significant increase in tissue metabolism and regeneration. It also induces angiogenesis [39].

Despite the reported clinical and radiographic success of VPT using various materials and techniques, histological assessments can best reveal the actual positive and negative effects of this treatment, and the actual pulp status with respect to inflammatory reactions, hard tissue formation, and the changes that occur in necrotic layers of tissue. Thus, this study aimed to review the histological results of VPT when undertaken using lasers.

## Methods

### Scope of the review

A comprehensive review of the histological results of VPT using lasers was undertaken, taking into account histological criteria for success and failure of VPT in both primary and permanent teeth. The influence of laser wavelengths and laser exposure parameters was considered, as these can make a drastic difference in treatment outcomes, and produce controversial results.

### Search strategy

An electronic search of the Scopus, MEDLINE, Web of Science, and Google Scholar databases covering the period 2000–2022 was undertaken by two independent investigators. The search strategy used different combination of keywords including laser, Er:YAG, Er,Cr:YSGG, Nd:YAG, diode, low-level laser therapy, CO_2_, pulp, vital pulp treatment, vital pulp therapy, direct pulp cap, pulpotomy, apexogenesis, and similar phrases. All the essential and relevant studies were included in the review. In addition, reference list mining of selected papers was undertaken to locate further articles and enrich the findings. Articles written in languages other than English were only included if the abstract was in English and contained all the required information on laser parameters and histological outcomes.

### Inclusion and exclusion criteria

**Fig. 1:**
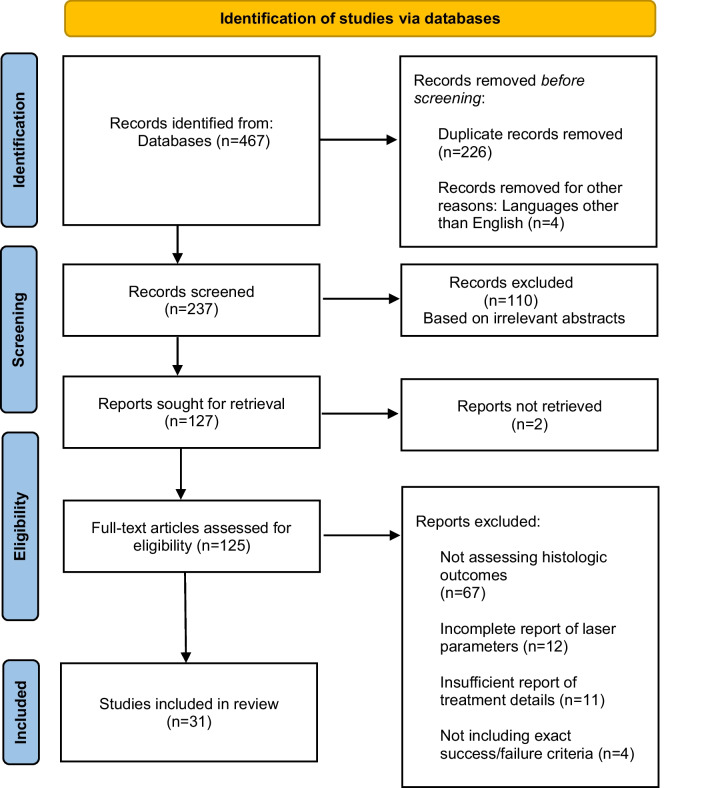
Source selection flowchart following PRISMA principles

**Fig. 2:**
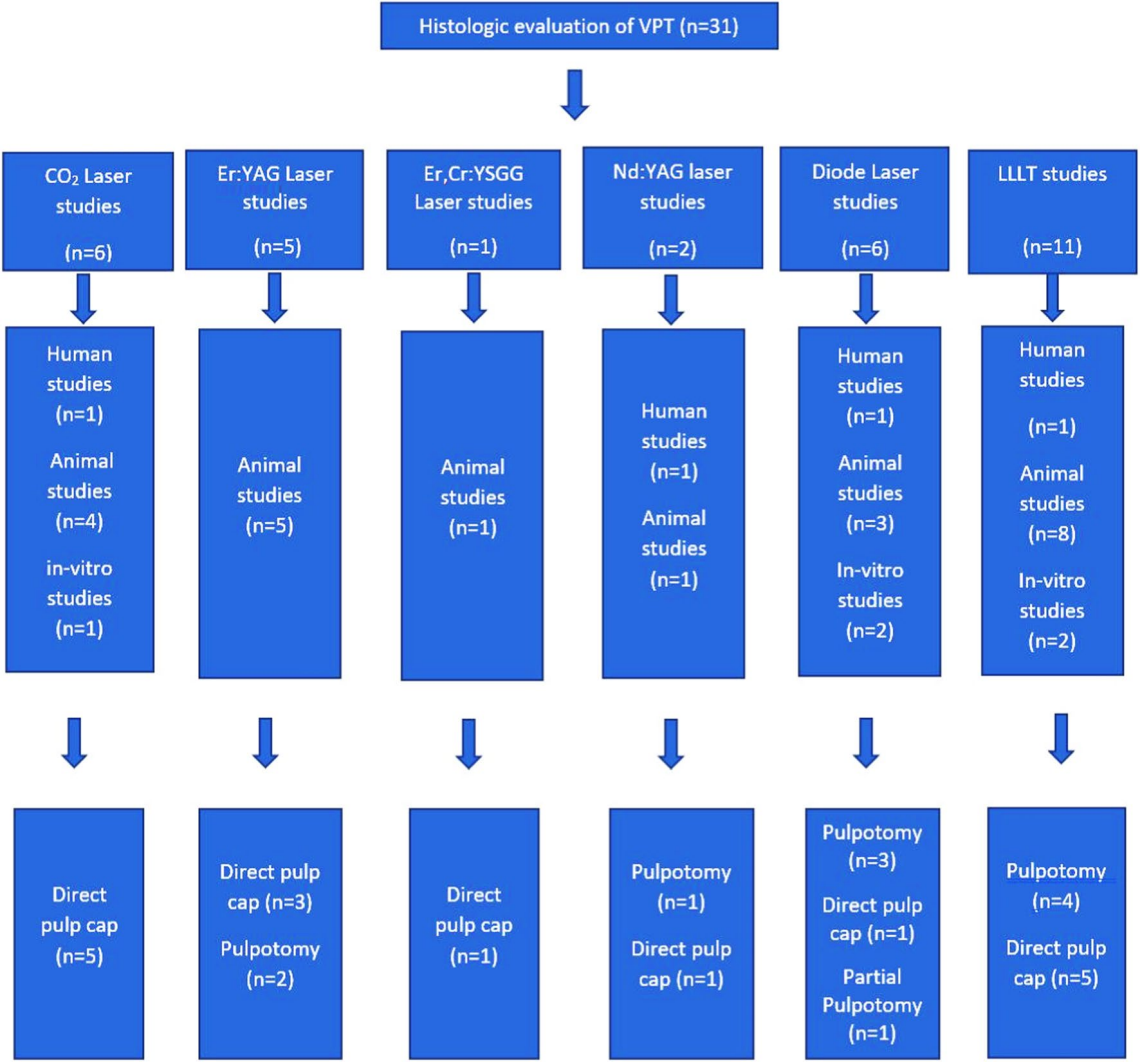
Classification of the included articles based on the type of pulp therapy, study, and laser used

Only human and animal in vivo studies on laser applications in vital pulp therapy were included where the laser parameters and criteria for success and failure criteria were mentioned explicitly. Additionally, in vitro studies were included if they evaluated any potential histological results of laser irradiation on dental pulp cells, including changes in cell morphology, tissue structure, gene expression, protein synthesis, or other relevant histological parameters. All important histologic outcomes (both positive and negative) were considered as being of interest. Since the study concentrated on histologic assessments of the outcomes of VPT, studies that were limited to clinical and radiographic findings were excluded. Source selection according to the Preferred Reporting Items for Systematic Reviews and Meta-Analyses (PRISMA) and the classification of selected papers are presented in Figs. 1 and 2, respectively.

## Results

Numerous studies have evaluated the application of different laser types for VPT and compared their results with conventional treatments. Some studies evaluated the success or failure of treatments based on histological criteria, while others assessed treatment outcomes using clinical and radiographic examinations. The present study was limited to collating studies using lasers for VPT that assessed outcomes using histological analysis.

When laser light is absorbed by tissues, photothermal effects can include tissue ablation, vaporization, and hemostasis. Vaporization results in cleavage of molecular bonds, bacterial destruction, and antibacterial actions [21]. In line with this, some studies reported promising results with respect to reduction of bacterial load following irradiation using lasers of different types [40–42]. Laser irradiation at high power densities can create a superficial layer of coagulation necrosis. This layer is compatible with the underlying tissue, and prevents direct contact of restorative or pulp capping materials placed onto it with the underlying pulp tissue [43].

**Fig. 3:**
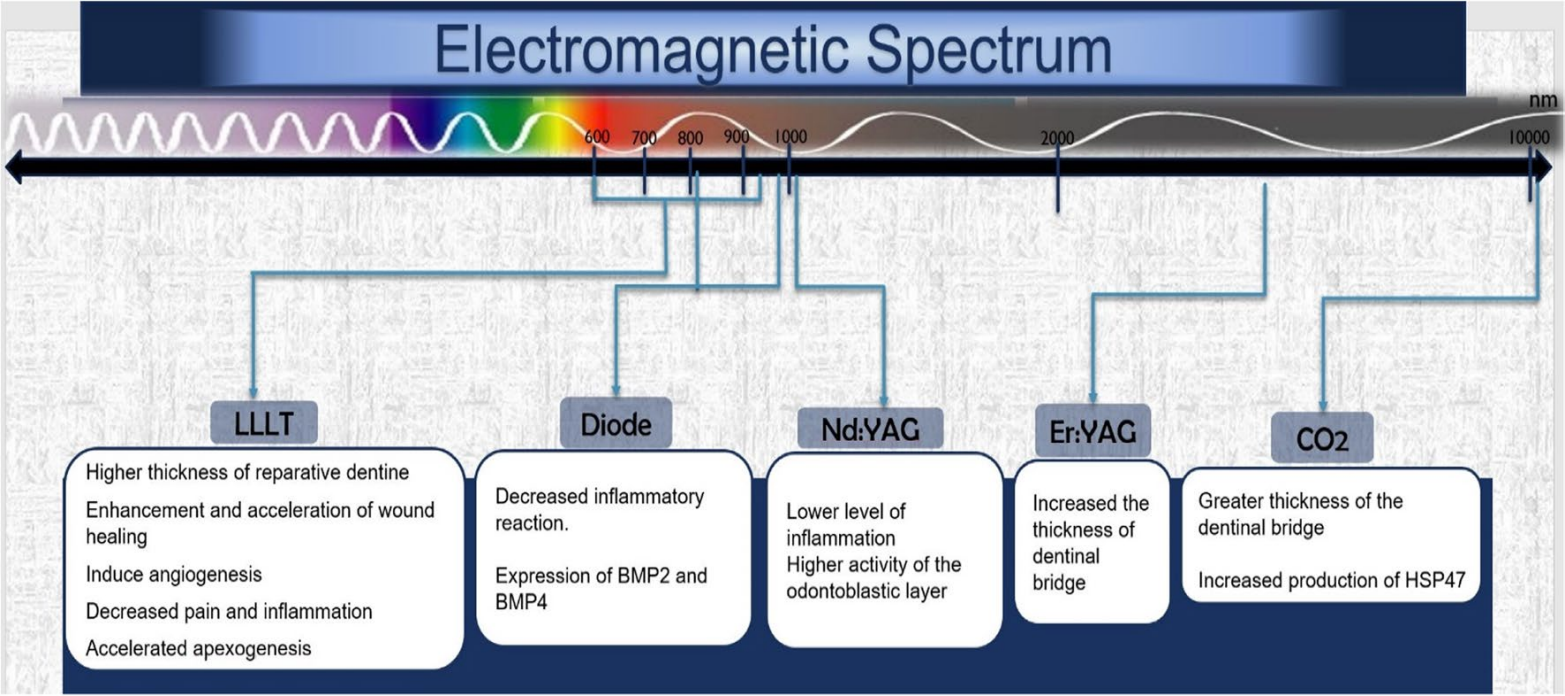
The histologic effects of various laser types on pulp tissue

Moreover, PBM laser irradiation can cause non-thermal effects that lead to increased formation of calcified nodules, enhanced alkaline phosphatase activity, and increased synthesis of collagen and osteocalcin [44]. The proven or suggested effects of various lasers on dental pulp are presented in Fig. 3.

### CO_2_ laser

Irradiation of dental pulp cells with the CO_2_ laser significantly promotes pulpal calcification process and also increases expression of heat shock protein-47 (HSP47), which plays an important role in collagen synthesis [45].

Histological analyses regarding the application of the CO_2_ laser prior to placing a pulp capping material have yielded variable results. When adhesive agents are placed, using the CO_2_ laser prior to pulp capping treatment causes mild tissue disorganization or pulpal inflammation and might delay dentinal bridge formation [23, 46, 47]. However, when pulp capping with calcium hydroxide is used following irradiation with the CO_2_ laser, dentinal bridge formation is enhanced, and the bridge achieves a significantly greater thickness [48].

Altering the intensity and duration of exposure alters the treatment outcomes for the CO_2_ laser, in a similar pattern to other laser types. Exposures using a lower output power accelerated pulpal healing and the formation of a dentinal bridge [47]. Extended exposure times (60 s) caused a 20% reduction in cell viability, an effect not seen with shorter exposures (20 and 40 s), while irradiation for 40 s increased the formation of mineral [45].

### Er:YAG laser

Some studies compared the Er:YAG laser with conventional rotary drills for cavity preparation in VPT [24, 49, 50]. Unlike high-speed drills, this laser did not create dentinal debris. Hence, when considering the possible infectivity of such debris, especially in carious teeth, the pulp environment may remain sterile when the laser is used to access the pulp chamber [24].

Using the Er:YAG laser instead of high-speed drills increased the thickness of dentinal bridge when pulp capping with calcium hydroxide or Biodentine™ [24, 50]. However, there was no significant difference in dentinal bridge formation or in the severity of inflammatory reactions when pulp capping was undertaken using mineral trioxide aggregate (MTA) cement after Er:YAG laser treatment [51].

As with other lasers, increasing the laser exposure parameters can significantly affect the results. For example, when the Er:YAG laser was used for pulpotomy with a pulse energy of 34 mJ, no inflammation or resorption in the pulp tissue was seen, whereas at higher pulse energies (68 and 102 mJ), moderate to severe pulpal inflammation was seen in 90% of cases [52]. One study reported that using the Er:YAG laser with 200 mJ pulses at 20 Hz for VPT resulted in thicker dentinal bridge formation after 8 weeks when compared with 100 mJ at 20 Hz. However, after 4 weeks the thickness of dentinal bridge was higher in 100 mJ irradiation group [49].

### Er,Cr:YSGG laser

Most studies on erbium lasers used the Er:YAG laser, and thus studies employing the Er,Cr:YSGG laser for VPT are limited. Pulp capping with the Er,Cr:YSGG laser has been reported to give superior results compared with formocresol, with a more uniform odontoblastic layer, and less inflammation, necrosis, and resorption [53].

In general, using lasers in the erbium family of lasers should cause a limited pulpal temperature rise when used in non-contact mode with associated cooling from an air and water spray [13, 54]. Both Er:YAG and Er,Cr:YSGG lasers have adjustable air and water spray flow rates, but the latter offers a finer degree of control by the user from the laser control panel, giving better tissue temperature control [13, 54].

Due to stronger absorption in water, the Er:YAG laser has a shallower penetration depth compared with the Er,Cr:YSGG laser (100 μm vs. 300 μm) [54]. Thus, the disinfecting and hemostatic effects of erbium lasers are less than those of other commonly used laser types such as diode, CO_2_, and Nd:YAG lasers, due to the shallower penetration depth in soft tissues [54].

### Nd:YAG laser

Few studies were found regarding the use of the Nd:YAG laser in VPT. A study conducted by Odabaş et al. that included histological assessments as well as clinical and radiographic evaluations found that the Nd:YAG laser did not show any significant superiority to formocresol [55].

In another study, pulp tissue was irradiated with the Nd:YAG laser in either contact or non-contact mode, before applying either calcium hydroxide paste or tin foil as pulp capping agents. Irradiation of pulp tissue with the Nd:YAG laser before application of calcium hydroxide, resulted in less inflammation and a higher activity of the odontoblastic layer compared with the application of this laser before placement of tin foil [56]. This finding emphasizes the importance of using a suitable pulp capping agent after laser therapy.

### Diode lasers

Table 1 summarizes the histologic effects of various diode lasers on dental pulp tissue.

**Table 1:** Comparison of histological effects of diode lasers compared to traditional therapies in treatment of vital pulp at 12 months

First author	Study type	Number of teeth	Treatment type	Materials used in vital pulp therapy	Laser parameters	Follow-up duration	Findings
Cannon et al. [57]	Animal	36 primary premolars and molars	Pulpotomy	FS+ZOE+RMIFC+ZOE+RMILaser+ZOE+RMGI	Diode laser980 nm, 3 W,120 s, 300 µm fiber, 100 ms intermittent pulses	4 weeks	Inflammatory response showed a significant difference among experimental groups. Laser, FC, and FS showed slight, slight to moderate, and moderate response, respectively.
Matsui et al. [58]	In vitro			Non lased (control) Laser 1 W	Diode laser Ga-Al-As 810 nm 1 W Non-contact (5 cm) 500 s, Optical fiber diameter: 600µm	28 days	Laser irradiation significantly increased the formation of calcified nodules. Laser irradiation significantly increased the BMP2 production after 48 and BMP4 production after 72 hours. ALP showed significantly more activity in laser group.
Yilmaz et al. [59]	in vitro	100 primary first molars (free of caries) (after extraction)		Hard setting CH Light cure CH (650 mW/cm^2^), 20 s Light cure CH (1000 mW/cm^2^), 20 s Light cure CH (4500 mW/cm^2^), 20 s Light cure dentine bonding (650 mW/cm^2^) Light cure dentine bonding (1000 mW/cm^2^) Light cure dentine bonding (4500 mW/cm^2^) Diode Laser (0.7 W) (20 s) Diode Laser (1 W) (20 s) Diode Laser (1.5 W) (20 s)	Diode 810 nm 0.7, 1, 1.5 W continuos mode, 1–2 s, 400 μm, Non-contact mode (1 mm)		Lowest temperature rise was reported in the light cure CH (650 mW/cm^2^) group. Laser groups (1 W, 1.5 W) showed greater temperature rise than other groups. Sealing integrity was incomplete in lased groups, but complete in other groups.
Al agele et al. [60]	Animal	40 incisors of rabbits	Partial pulpotomy	MTA (control) Laser+MTA BMP-7+MTA Laser+BMP+MTA	Diode laser 808 nm, 10 W, CW	1 week, 4 weeks	There was no significant difference between the BMP and BMP +Laser group.
Mareddy et al. [61]	Animal	42 premolars of dogs	Pulpotomy	Control 1-s laser (hemostasis)+IRM 3-s laser (hemostasis)+IRM 5-s laser (hemostasis)+IRM	Diode laser 810 nm, 2 W, 0.5 Hz, 1, 3, and 5 s	1 day, 1 week	Most regressive changes were related to 5 seconds of laser application.1 and 3-second were the best application times for diode laser pulpotomy as odontoblastic layer was intact in most of the specimens.
Sivadas et al. [62]	Human in vivo	24 primary teeth	Pulpotomy	FS+ZOE+GI (GC) Laser+ZOE+GI (GC)	Diode 810 nm, Continuus mode, 1.4 W, 5 s, Contact mode, Optical fiber diameter: 500 µm	30 days 45 days	There were no significant differences between the two groups with regard to the quality and location of dentine bridge formation, tissue reactions, and inflammatory responses.After 30 days, none of the groups showed necrosis. After 45 days, the laser group showed significantly more necrosis.

*DPC*, direct pulp cap; *CH*, calcium hydroxide; *FC*, formocresol; *ZOE*, zinc oxide eugenol; *IRM*, intermediate restorative material; *RMGI*, resin modified glass ionomer; *MTA*, mineral trioxide aggregate; *GI*, glass ionomer; *FS*, ferric sulfate, *CW*, continuous wave, *BMP*, bone morphogenic protein

Diode laser exposure can decrease inflammatory reactions following VPT. Histologically, pulpotomy with diode laser causes significantly less inflammation compared with formocresol [57]. According to an in vitro study on human pulp cells, application of the diode laser at a power of 1 W significantly increased the formation of calcified nodules and the expression of BMP4, BMP2, and alkaline phosphatase, compared with 0.5 W [58].

Since the seal created by laser on the soft tissue in the process of pulp capping is insufficient, a pulp capping agent is necessary after diode laser irradiation [59]. In histological assessments, application of a diode laser prior to the use of pulp capping agents such as MTA and BMP7 did not cause a significant change in the results [60].

As with other laser types, different exposure parameters for the diode laser can yield different outcomes. Average output powers higher than 0.7 W can cause hazardous pulpal temperature rises [59], and prolonged exposure times (> 3 s) can cause regressive changes in pulp tissue [61]. Based on a study by Sivadas et al., there was not any significant difference between ferric sulfate and diode laser in pulpotomy of primary teeth, although the quality of formed dentinal bridge was better in lased groups [62].

### Low level laser therapy (LLLT)

**Fig. 4:**
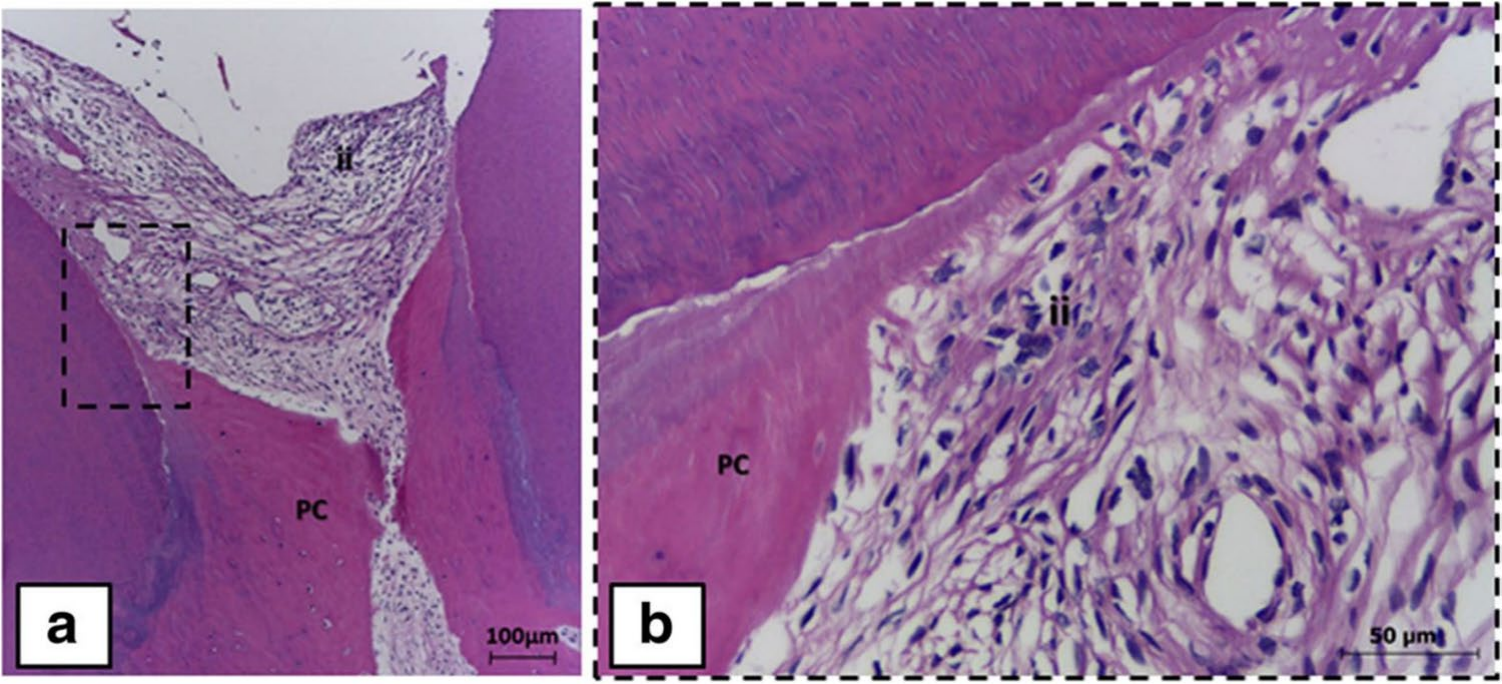
Low-level laser therapy (LLLT)+zinc oxide and eugenol group. **a** Loose connective tissue with moderate inflammatory infiltrate (ii) and pulp calcification, better observed in higher magnification. **b** Hematoxylin and eosin staining. Scale bar of 100 (**a**) and 50 μm (**b**). Reproduced with permission from the publisher [36]

Table 2 summarizes the available data of LLLT for PBM in VPT. PBM has a long history of use for enhancing wound healing and boosting regenerative treatments. Some histologic changes after LLLT are shown in Fig. 4. PBM can reduce inflammation and enhance the proliferation of stem cells [35–37]. At the level of the cell, biological effects of PBM include increasing adenosine triphosphate (ATP) synthesis by the mitochondria, accelerating type I and III collagen synthesis by fibroblasts, and enhancing wound healing [37, 63].

**Table 2:** Comparison of histological effects of LLLT for photobiomodulation compared to traditional therapies in vital pulp therapy

First author	Study type	Number of teeth	Treatment type	Materials used in vital pulp therapy	Laser parameters	Follow-up duration	Findings
Bidar et al. [15]	Animal in vivo	25 premolar teeth of dogs	DPC	MTA+ GC cement LLRL (630 nm) + MTA+ GC cement LLIL (810 nm) + MTA+ GC cement LLRL (630 nm) + Gold foil+ GC cement LLIL (810 nm) + Gold foil+ GC cement	LLRL 630 nm, CW, 20 mW Non-contact (2 mm), 150 s, 7.5 J/cm^2^, Continuous mode, LLIL 810 nm, 50 mW, 1500 Hz, 50 s, Spot size of 0.2cm^2^, 6.25 J/cm^2 ^	2 months	The mildest and the most severe inflammatory reactions were reported in LLIL+MTA and LLRL+gold, respectively. Hard tissue formation in groups with application of MTA was 100% complete. The odontoblastic layer existed in 100% of teeth in MTA/LLRL+MTA/LLIL+MTA and in 80% of teeth in LLRL+gold and LLRL+gold. None of the groups showed diffuse calcification. Necrosis did not exist in groups with MTA application, although partial necrosis was reported in LLRL+gold and LLIL+gold.
Marques et al. [36]	Human in vivo	20 mandibular primary molars	Pulpotomy	FC+ZOE+IRM+RMGI CH_+_ IRM+RMGI Laser+ZOE_+_ IRM+RMGI Laser+CH_+_ IRM+RMGI	LLLT In-Ga-AlP 660 nm, 10 mW, 2.5 J/cm^2^, 10 s, continuous mode, 50–60 Hz, Optical fiber diameter: 320 µm, Focus beam of 0.04 cm^2^	Mean time: 11.73 months	The barrier formation was just seen in groups with application of CH. The lowest level of inflammation was reported in Laser+CH+IRM+RMGI group. CH group was significantly superior in terms of hard tissue barrier, odontoblastic layer and dense collagen fibers.The highest degree of internal resorption was reported in FC group.
Pretel et al. [39]	Animal	45 permanent premolar teeth of monkeys	DPC	CH+amalgamLaser 688 nm+CH+amalgam Laser 785 nm+CH+amalgam	LLLTGa-Al-As semi conductor diode laser 688, 785 nm, Continuous mode, 50 mW, 20 s, 255 J/cm^2^, 2 J, Tip’s area: 0.00785 cm^2^	7 days, 25 days, 60 days	After 25 days, the highest thickness of reparative dentine was in the Laser 785/Laser and 688/CH groups. After 60 days, the thickness of reparative dentin in lased groups was higher than the non-lased group which was significant for 785 nm laser group.
Prabhakar et al. [64]	Animal	20 primary premolar teeth of dogs	Pulpotomy	LLLT+ZOE base FC+ZOE base	LLLT Diode laser 660 nm, 36 mW, Non-contact mode, 4 J/cm^2^	1 week	The inflammatory response was significantly more severe in FC group rather than in the LLLT group.
Shalash et al. [65]	Animal	48 teeth in dogs	DPC	CH+GI CH+GI+LLLT(PBM)	LLLT(PBM) Soft laser870 nm, 50 mW, CW	1,8,16 weeks	At 16 weeks, the formed dentinal bridge was significantly thicker in the PBM group. At 1 week, the inflammatory response in non-lased group was more intense.
Alsofi et al. [66]	Animal	24 anterior teeth of rabbits	DPC	Laser+endosequence root repair material (ERRM)+GI (after 2 weeks) ERRM+GI (after 2 weeks) Laser+endo sequence repair material (ERRM)+GI (after 2 months) ERRM+GI (after 2 months)	LLLT Diode laser 980 nm, 0.25 W, Contact mode, 90 s, 1.25 J/cm^2^	2 weeks, 2 months	After 2 weeks/2 months, the inflammatory response in laser groups was mild. while in non-lased groups it was severe, and then decreased to mild at 2 months. After 2 weeks/2 months, the odontoblastic layer was organized in a palisaded manner in the laser groups. After 2 weeks, the odontoblastic layer in non-lased groups was disorganized, but became palisaded after 2 months. Predentine thickness was significantly higher in laser groups.
Alharbi et al. [67]	Animal	80 teeth	DPC	LLLT+ERRM (endoseguence)(fast set) LLLT+ERRM (endoseguence) (regular set) No treatment (control) ERRM	LLLT Diode laser 870 nm, 100 mW, 1.08 J/cm^2^, 150 s	2 weeks, 2 months	The LLLT groups had greater reparative dentin area than the non-lased group.The dentine bridge quality improved significantly after two months compared to two weeks.
Ferriello et al. [68]	In vitro		Laser treatment	Control (fresh culture medium) CH Adhesive resin Control+laserCH+laser Adhesive resin+laser	LLLT Diode Laser 680 nm, 60 s, 4 J/cm^2^, Non-contact mode (1 cm)	1,3,5,7 days	Laser irradiation slightly increased the proliferation rate but the effect was not significant.
El Nawam et al. [69]	In vitro	40 tooth slices of human third molar	Laser treatment	No laser treatment 660-nm diode (1 J/cm^2^) 660-nm diode (3 J/cm^2^) 810-nm diode (1 J/cm^2^) 810-nm diode (3 J/cm^2^)	LLLT (PBM)Diode laser (Ga-Al-As)660, 810 nm, 1–3 J/cm^2^	7 days	Irradiation 660 nm @ 1 J/cm^2^ caused significant upregulation of VEGF, VEGFR2, and odontogenic genes [DSSP (6.1-fold), DMP-1 (3-fold), BSP (6.7-fold)] Irradiation 810 nm @ 3 J/cm^2^ caused significant upregulation of odontogenic genes [DSPP (2.5-fold), DMP-1 (17.7-fold), BSP (7.1-fold)]
Bahman et al. [70]	Animal in vivo	36 immature permanent anterior and premolar teeth	Pulpotomy (Apexogenesis)	CH+Amalgam CH+Amalgam+Ga-Al-As (PBM)	LLLT (PBM) Ga-Al-As 810 nm, 0.3 W, 4.2 J/cm^2^, 9 s, CW	14 days	After 7 days, PBM enhanced the amount of deposited dentine. The combination therapy of PBM and pulpotomy with CH accelerated apexogenesis in immature permanent dog teeth
Fekrazad et al. **[**71**]**	Animal	40 immature permanent teeth	Pulpotomy (Apexogenesis)	MTA+Amalgam+Ga-Al-As laser MTA+Amalgam	LLLT Ga-Al-As 810 nm, 0.3 W, 4 J/cm^2^, 9 s, Optical fiber diameter: 0.9 cm	14 days	Irradiation with 810-nm laser accelerated the rate of dentinogenesis in apexogenesis of immature permanent teeth

*DPC*, direct pulp cap; *CH*, calcium hydroxide; *FC*, formocresol; *ZOE*, zinc oxide eugenol, *IRM*, intermediate restorative material, *RMGI*, resin-modified glass ionomer, *LLRL*, low-level red laser, *MTA*, mineral trioxide aggregate; *LLIL*, low-level infrared laser; *ERRM*, endosequence root repair material, *GI*, glass ionomer, *FS*, ferric sulfate, *CW*, continuous wave, *PBM*, photobiomodulation

In the setting of VPT, laser irradiation alone for PBM without the application of a pulp capping agent will not yield the desired outcome [46, 72]. PBM alone [64] or prior to the application of pulp capping agents such as calcium hydroxide [36, 65], MTA [15], and endosequence root repair material [66, 67], can increase the thickness of the dentinal bridge and decrease inflammation following VPT. It also has been shown to accelerate the healing process, [15, 36, 39, 64, 66]. This could explain a lower rate of internal resorption in teeth subjected to laser therapy [36].

The extent of inflammation following pulpotomy is lower when PBM is used than with formocresol [64]. Several laser parameters (810 nm for 3 J/cm^2^; and 660 nm for 1 J/cm^2^) induce angiogenesis and dentinogenesis [69]. Regarding 660-nm wavelength, lower energy densities (1J/cm^2^) increase the expression of angiogenic and odontogenic genes, significantly, while higher energy densities (3 J/cm^2^) have such effect only on angiogenic genes. For 810-nm wavelength, higher energy densities (3 J/cm^2^) have stronger boosting effect on the expression of odontogenic genes in comparison with 1 J/cm^2^, while the effect on angiogenic genes remains the same for both energy densities [69]. 

Several studies focused on PBM effects rather than photothermal disinfecting and coagulation effects of diode lasers. PBM effects can be achieved by indirect irradiation of the dental pulp, for instance, through the buccal and lingual gingiva of permanent teeth with immature apices, following pulp capping with calcium hydroxide [70] and MTA [71] once every 2 days for 2 weeks. This was shown to significantly accelerate apexogenesis. In another study, after pulp capping and final restoration of teeth, PBM was applied immediately after restoration, and also at 48 and 96 hours later. After 16 weeks, the laser group showed a higher thickness of reparative dentine [65].

## Discussion

Several studies have assessed the efficacy of VPT with lasers, and reported variable results. The possible reasons for such variations are discussed below, in terms of the parameters that need to be taken into account when comparing the outcomes for VPT.

### Histological criteria

The criteria used in these studies included the following:

(I) Presence of inflammation: In many studies [15, 23, 24, 46, 47, 52, 62], the inflammatory responses were assessed, and graded either as an absence of inflammation or as mild, moderate, and severe inflammation. Scoring systems ranged from 0 to 4 [36, 57], 0 to 3 [24, 51–53, 55, 62], 1 to 4 [23, 39, 47, 60, 64, 66], or 1 to 3 [15, 51, 65]. Factors considered by different studies when evaluating inflammation included the presence of inflammatory cells and their distribution [23, 24, 46, 47, 51, 52, 60, 62, 66, 73], bleeding [24], micro-abscess formation [57], signs of acute or chronic inflammation [15, 51, 61, 65], and changes in pulp morphology [23, 47, 73]. In some studies, grading was based on the count of inflammatory cells, and particularly neutrophils/polymorphonuclear leukocytes (PMNs). For instance, many studies graded inflammation based on the number of PMNs as follows: mild: PMNs < 30, moderate: 30 < PMNs < 60, and severe: PMNs > 60 [15, 51, 53, 65], while others used the following scoring system: mild: PMNs < 10, moderate: 10 < PMNs < 25, and severe: PMNs > 25 [39, 64].

(II) Dentinal bridge formation (reparative dentine): Some studies classified the extent of dentinal bridge (reparative dentine) formation into the absence of visible bridge, incomplete bridge formation, or complete bridge formation [15, 23, 24, 46, 47, 65]. In other studies, direct, close, and moderate contact between the pulp capping agent and the pulp tissue was considered as a criterion [15, 51]. Some other studies rated the quality and morphology of the dentinal bridge in terms of its tubule pattern [15, 62], or its thickness [15, 51, 65].

(III) Changes in pulpal morphology (disorganization): Some studies assessed the presence of hyperemia [51, 53, 61], vascularization [36, 53], collagen fibers [36], necrosis [15, 51, 53, 61, 62], and the organization status of the odontoblastic layer at the exposure site [15, 36, 51, 60, 66].

(IV) Some studies assessed the expression of molecules such as DMP1 [46, 47], HSP47 [45, 46], and TGF-B1 [46, 47] that are indicators of reparative dentin formation using immunohistochemistry. DMP1 and HSP47 are expressed by odontoblasts or by odontoblast-like cells that are present beneath the newly formed reparative dentine layer [46].

(V) Presence of bacteria: Some studies evaluated the presence of bacteria in cavity walls, dentinal tubules, or pulp tissue [23, 24, 46, 47, 55, 73]. This assessment reveals possible entry of bacteria in the processes of caries removal or access cavity preparation [47], and also provides insight into antimicrobial effects of laser exposure, and the quality of the seal created by the pulp capping agent and restorative material.

### Duration of follow-up

The duration of follow-up varied from 0 days [52] to 4 months [51] in animal studies, from 1 day [45] to 28 days [58] for in vitro studies, and from 7 days [55] to 12 months [23] in human studies. The use of short time periods (days to weeks) does not align with clinical practice or with studies of VPT that use clinical and radiographic assessments. This prevents direct comparison of results.

### Factors related to laser parameters

As well as using different laser wavelengths, different laser exposure parameters were used within studies of the same laser type (operating mode, average power, pulse frequency, exposure time, power density, energy density, air/water or dry condition). Altering exposure parameters influences the nature of the laser effect on dental pulp tissue [33].

In terms of photothermal changes, using high laser pulse energies caused greater injury to the pulp and periodontal tissues, compared with lower energies, e.g., Er:YAG at 68 and 102 mJ/pulse rather than 34 mJ/pulse [52]. If excessively high laser exposures are used, infiltration of inflammatory cells, carbonization, and necrosis will occur in a dose-dependent manner [74], and this could lead to failure of VPT. Thus, it is important to choose laser parameters that achieve the appropriate level of thermal effects on the pulp tissue. For pulp capping, hemostatic effects are desirable, rather than ablation.

In order to prevent thermal damage, water irrigation can be used, and the average power, pulse frequency, and total duration of laser irradiation can be minimized [75]. As an example of exposure time, Mareddy et al. undertook pulpotomy with a diode laser at a power of 2 W, and found that laser irradiation for 1 to 3 s was ideal, but that regressive changes occurred when the laser exposure time extended to 5 s [61].

Appropriate laser parameter selection will ensure optimal coagulation of pulp tissue to form a thin necrotic layer on the residual pulp. This layer prevents the direct contact of the pulp tissue with the capping material, and inhibits irritant or toxic effects of the pulp capping agent [74]. The layer also acts as a focus for the migration of inflammatory cells and fibroblasts, which participate in dentinal bridge formation [27]. Excessive laser output powers, energy density or exposure time will increase the thickness of the denatured layer, and inhibit or delay the formation of a dentinal bridge [23, 46, 47, 73]. In other words, the thicker this layer, the longer it will take for reparative dentine to form. If carbonized tissue is present, it could physically retard dentine formation, and hence it will take longer for a dentine bridge to form [46]. When considering the choice of laser parameters, it is important to bear in mind that dental pulp tissue is confined to the dentine of the coronal tooth structure, and heat generated in that tissue is less able to be removed by normal blood flow than is in the case in oral mucosa or skin. Hence, while dentine has some heat sinking abilities [47, 76, 77], at the same laser settings, a thicker coagulation necrosis layer will be created when the pulp is lased than when the same settings are used on oral mucosa or skin [78].

When the pulpal temperature increases by more than 5° (10 °F), pulpal injury occurs, and temperature rises above 11.1 °C (20 °F) lead to necrosis [79]. The influence of laser parameters on VPT outcomes was seen in the study by Ogisu et al., where a complete dentine bridge was formed in some cases following PBM, but not when moderate or high intensity laser settings were used [47]. This was despite the fact that a coolant was also used, but this may not have been sufficient to prevent thermal damage at high laser exposures.

The choice of laser operating mode is important, as this alters the ability of the tissue to cool, the thickness of any coagulation layer that is formed, and whether carbonization occurs. Shoji et al. reported that the pulpal reaction to the CO_2_ laser was influenced by the irradiation mode and time [78]. In this regard, Suzuki et al. used a CO_2_ laser operating in super-pulse mode. In this mode, a series of high amplitude radiation pulses are emitted that have a short duration [23, 46, 73]. This lowers carbonization of the soft tissues and prevents damage to deeper parts of the pulp due to accumulation of heat.

The depth and extent of photothermal tissue change caused by lasers in the dental pulp will vary depending on the laser wavelength that is chosen, from 0.03 mm for the Er:YAG laser to 0.1 mm for the CO_2_ laser, to 3–5 mm for the Nd:YAG and near-infrared diode lasers, depending on the delivery system and whether contact or non-contact tips are used. This explains their different biological effects [18]. Controlling the extent of photothermal tissue change is a key task when using Nd:YAG and diode lasers, and can be challenging because initiation of a plain fiber tip end occurs during treatment as the tip comes into contact with blood, and carbonization occurs. This increases the tip temperature, causing a “hot tip” action. The hot tip can itself then cause thermal injury and coagulation. Adjustment of the laser parameters and correct tip selection and use is particularly important [55].

For PBM, while a range of lasers have been used, those in the visible red spectrum (600 to 700 nm) applied at an energy density of 0.5 to 4 J/cm^2^ have the greatest effect on cell proliferation, for example, 675 nm at 2 J/cm^2^ energy density. On the other hand, 810 nm at 10 J/cm^2^ had an inhibitory effect on cell proliferation [35, 36]. Irrespective of the laser type, PBM causes a photo-bioactive reaction and induces healing during the inflammatory phase [47] by promoting remodeling [15]. It increases the number of fibroblasts and boosts ATP synthesis [36] as well as the production of collagen [47].

### Misdiagnosis of the sound pulp

Reported failures for VPT in the literature can reflect a range of other factors, including clinical errors in diagnosis and patient selection. Incorrect diagnosis of a pulp with chronic inflammation or infection can lead to treatment failure [55]. This is particularly true for pediatric patients, since their verbal responses to questions regarding symptoms are not highly reliable, putting greater emphasis on the experience and knowledge of the clinicians in forming their diagnosis [80].

### Difference in samples

The types of pulp situation present when VPT is undertaken can affect the outcome when using lasers or other treatment approaches. In the study by Raslan and Wetzel [81], dental pulps that were exposed due to trauma showed less inflammatory reactions than those exposed due to deep caries. Most histologic studies of VPT, especially those using animals, began with a baseline of sound asymptomatic teeth with no clinical and radiographic pathology [15, 39, 64]. This is not a realistic starting point for VPT, which limits the clinical application and generalizability of the results to sound teeth. Few studies assessed VPT using teeth with dental caries [36, 55, 62].

When considering the use of lasers in VPT, laser safety elements must be taken on board, to lower risk and prevent harms associated with the application of lasers. These include the need for clinical staff and patients to wear suitable eye protection that fully protects them from direct and indirect exposure during laser application to the teeth [82]. Secondly, precautions should be taken to keep flammable materials away from the laser beam to avoid ignition and surgical fires [82]. This requires special attention when dental laser treatments are being performed on patients receiving nitrous oxide and oxygen mixtures, which support combustion. Careful attention must be paid to the risks of the beam being reflected and inadvertent damage to adjacent teeth and oral soft tissues [18, 78, 82].

###  Limitations, gaps, and recommendations

A draw-back to the routine use of lasers for VPT procedures such as pulpotomy is the cost of the equipment [83].

Human studies where teeth are extracted for histological analysis are challenging because of issues of noncompliance and high dropout rates, as well as the impediments regarding the timing of tooth extraction. These result in small sample sizes and relatively short follow-up periods [36].

Ideal or “gold standard” histologic studies to evaluate techniques and materials for VPT will typically be in vitro or animal studies. These allow control of the timing of interventions and overcome issues of compliance. Such studies can address, at least in part, the lack of information on the human application of lasers in VPT.

When PBM has been applied, most studies have used only a single dose application and did not continue PBM after restoring the tooth. More work is needed to assess repeated exposures for PBM in VPT. It is well known that repeated intermittent laser irradiation has optimal PBM actions for promoting wound healing, such as in the setting of human skin ulcers [15].

Histologic effects of laser application depend on the laser type and laser parameters as well as types of cells or tissues being studied [35].

In the present study, while the time frame of 2000–2022 was chosen to capture relevant and up-to-date information, it is acknowledged that research from earlier years may hold further insights to the topic of lasers in VPT.

There are limited studies which compare different laser wavelengths for the same procedure. Therefore, future studies should evaluate different types of lasers when used for the same VPT procedure [62]. This is particularly important for diode lasers, where a wide range of wavelengths exist.

Few histological studies have follow-up periods of more than 2 months. Therefore, further work should use longer time intervals to evaluate lasers used for VPT [69].

## Conclusion

Laser technology has proven valuable in endodontics and vital pulp therapy for both diagnostic and therapeutic purposes. Choosing appropriate exposure parameters is crucial for VPT. For lasers with photothermal actions, histological evaluations of VPT can reveal if the coagulation produced is excessive. For PBM, utilizing appropriate settings ensures that there is a worthwhile reduction in pulpal inflammation while enhancing the thickness of reparative dentine and the dentinal bridge. It should be noted that laser irradiation alone is inadequate to achieve the desired outcomes of VPT without the accompanying use of appropriate pulp capping materials.
